# Contribution of voltage-gated sodium channel β-subunits to cervical cancer cells metastatic behavior

**DOI:** 10.1186/s12935-019-0757-6

**Published:** 2019-02-15

**Authors:** Ana Laura Sanchez-Sandoval, Juan Carlos Gomora

**Affiliations:** Departamento de Neuropatología Molecular, División de Neurociencias, Instituto de Fisiología Celular, Universidad Nacional Autónoma de México, 04510 Mexico City, Mexico

**Keywords:** Voltage-gated sodium channel β-subunits, Cervical cancer, Proliferation, Migration, Invasion, Cell-adhesion molecule

## Abstract

**Background:**

Voltage-gated sodium (Na_V_) channels are heteromeric proteins consisting of a single pore forming α-subunit associated with one or two auxiliary β-subunits. These channels are classically known for being responsible of action potential generation and propagation in excitable cells; but lately they have been reported as widely expressed and regulated in several human cancer types. We have previously demonstrated the overexpression of Na_V_1.6 channel in cervical cancer (CeCa) biopsies and primary cultures, and its contribution to cell migration and invasiveness. Here, we investigated the expression of Na_V_ channels β-subunits (Na_V_βs) in the CeCa cell lines HeLa, SiHa and CaSki, and determined their contribution to cell proliferation, migration and invasiveness.

**Methods:**

We assessed the expression of Na_V_βs in CeCa cell lines by performing RT-PCR and western blotting experiments. We also evaluated CeCa cell lines proliferation, migration, and invasion by in vitro assays, both in basal conditions and after inducing changes in Na_V_βs levels by transfecting specific cDNAs or siRNAs. The potential role of Na_V_βs in modulating the expression of Na_V_ α-subunits in the plasma membrane of CeCa cells was examined by the patch-clamp whole-cell technique. Furthermore, we investigated the role of Na_V_β1 on cell cycle in SiHa cells by flow cytometry.

**Results:**

We found that the four Na_V_βs are expressed in the three CeCa cell lines, even in the absence of functional Na_V_ α-subunit expression in the plasma membrane. Functional in vitro assays showed differential roles for Na_V_β1 and Na_V_β4, the latter as a cell invasiveness repressor and the former as a migration abolisher in CeCa cells. In silico analysis of Na_V_β4 expression in cervical tissues corroborated the downregulation of this protein expression in CeCa vs normal cervix, supporting the evidence of Na_V_β4’s role as a cell invasiveness repressor.

**Conclusions:**

Our results contribute to the recent conception about Na_V_βs as multifunctional proteins involved in cell processes like ion channel regulation, cell adhesion and motility, and even in metastatic cell behaviors. These non-canonical functions of Na_V_βs are independent of the presence of functional Na_V_ α-subunits in the plasma membrane and might represent a new therapeutic target for the treatment of cervical cancer.

**Electronic supplementary material:**

The online version of this article (10.1186/s12935-019-0757-6) contains supplementary material, which is available to authorized users.

## Background

Cervical cancer (CeCa) is the fourth most common cancer in women worldwide, with more than 500,000 new cases each year and an estimated 260,000 deaths, of which approximately 87% occur in developing countries [1]. Despite the implementation of vaccination against Human Papillomavirus infection, it will take decades before the impact on the cervical cancer incidence became clear, giving the natural history of this disease [2]. Therefore, it is necessary to better understand the biology of this type of cancer in order to improve the early detection and treatment.

Voltage-gated sodium (Na_V_) channels are heteromeric transmembrane proteins that consist of a single pore forming α-subunit associated with one or more auxiliary β-subunits (Na_V_βs) [3]. These channels are known to be responsible for action potential generation and propagation in excitable cells, but they are also widely expressed and upregulated in a variety of human cancer types, including breast, prostate, colon, ovarian, lung, gastric, melanoma, astrocytoma and uterine cervix, where the Na_V_ α-subunit activity has been associated mainly, but not exclusively, with cell motility and invasiveness [4–14]. So far, four Na_V_βs (Na_V_β1–Na_V_β4) have been described. These are multifunctional type I transmembrane proteins that modulate Na_V_ α-subunit gating, localization and transit to the plasma membrane. Additionally, they possess a V-type immunoglobulin repeat in the extracellular domain similar to the family of neural cell adhesion molecules (CAMs), thus, their role as CAMs in a trans-homophilic and trans-heterophilic way, and with other molecules, in presence or absence of the α-subunits has been described in numerous reports and in different expression systems [15–22].

The role of Na_V_βs in cancer has also been studied, although not as widely as for the α-subunits. In prostate cancer (PCa), it has been found that the overexpression of Na_V_βs is associated with the increasing metastatic potential of PCa cell lines [23], and when overexpressing Na_V_β2 in LnCaP cells, the cells acquired a fibroblastic-like morphology, became more invasive and had enhanced migration in vitro; in contrast, these cells had a reduced tumor volume in vivo [24, 25]. On the other hand, the overexpression of Na_V_β3 in Saos-2 (osteosarcoma) and T98G (glioblastoma) cells, seems to activate an apoptotic pathway mediated by the tumor suppressor protein p53 [26]. However, most of the studies have been done in breast cancer, where it has been demonstrated that Na_V_β1 is overexpressed in the low metastatic MCF-7 cell line vs highly metastatic MDA-MB-231 cells [27]. Overexpressing Na_V_β1 in these last cells produced a drastic reduction on in vitro transwell migration and an increase in cellular adhesiveness. In contrast, MDA-MB-231 cells stably transfected with Na_V_β1 produced larger tumors in an in vivo model compared with those generated by wild type MDA-MB-231 cells. In addition, overexpression of Na_V_β1 induced a reduction in caspase activity in these cells [28]. More recently, the potential role of Na_V_β4 as a metastasis-suppressor gene has been described in breast cancer cells, associated with the small GTPase RhoA activity and therefore with the cytoskeleton remodeling during migration and invasiveness [29].

We have previously demonstrated the overexpression of Na_V_ α-subunits in cervical cancer biopsies vs non-cancerous cervix, and furthermore, we reported the functional expression of these proteins in primary cultures derived from human cervical cancer biopsies and their contribution to the cell migration and invasiveness. Specifically, Na_V_1.6 showed to be the most overexpressed α-subunit and the one responsible of the augmented invasive potential as the specific blockade of this channel was able to diminish the invasive potential with the same effectiveness as the blockade of all the Na_V_ α-subunits by TTX [14, 30]. In the present work, we investigated the electrophysiological activity of Na_V_ channels and the expression of Na_V_βs in three cervical cancer cell lines (HeLa, SiHa and CaSki) as well as their contribution to three metastatic cell behaviors: in vitro proliferation, transwell migration and invasiveness.  

## Methods

### Cell lines and cell culture

Cervical cancer cell lines SiHa, CaSki (both HPV-16 positive) and HeLa (HPV-18 positive) were grown in DMEM supplemented with 10% fetal bovine serum (FBS), 100 U/ml penicillin, and 100 μg/ml streptomycin at 37  °C in a humidified 5% CO_2_ incubator. All cell culture reagents were purchased from Gibco-Thermo Fisher Scientific (Waltham, MA). The three cell lines were authenticated by short tandem repeat profiling prior to the experiments (data not shown).

### Overexpression and downregulation of Na_V_βs expression

Plasmids containing each of the Na_V_βs from rat were kindly donated by Dr. L. Isom (University of Michigan). Overexpression of each Na_V_β in CeCa cells was achieved by transient transfections using the JetPEI transfection reagent (PolyPlus Transfection; Illkirch, France), according to the manufacturer’s instructions. On the other hand, downregulation of Na_V_βs expression was performed using two or three different predesigned siRNAs (Sigma-Aldrich; St. Louis, MO) for each Na_V_β (100 nM each); in all cases, we chose the one with the best efficiency. Optimal siRNA transfection conditions were stablished previously by using 50 nM of siGLO Green Transfection Indicator (Sigma-Aldrich), a fluorescent siRNA for determining transfection efficiency. Efficiency of both experimental strategies to manipulate the expression levels of each Na_V_β was evaluated by RT-PCR and western blot (see Additional file 1).

### Electrophysiology

Detailed methods for whole-cell patch-clamp recordings and protocols have been previously described [14, 30]. Sodium currents of native or transiently transfected CeCa cells were recorded at room temperature (20–23 °C) using an Axopatch 200B amplifier, a Digidata 1320A/D converter, and the pCLAMP 10.0 software (Molecular Devices; Sunnyvale, CA). As a positive control for sodium currents, we used transiently transfected CeCa cells with the Na_V_1.6 channel.

### Standard PCR (RT-PCR)

The Na_V_βs mRNA expression levels of CeCa cell lines were assessed by RT-PCR with specific primers for each β-subunit and β-actin as control (Table 1) as described before [30]. Briefly, total RNA of each cell line was isolated with Trizol reagent (Invitrogen; Carlsbad, CA) and RT-PCR reactions were performed in a final volume of 25 µl using the Super-Script One-step RT-PCR kit (Invitrogen) with 250 ng of total RNA, 0.5 µl of enzyme mix, 0.2–0.5 µM of each primer, and 12.5 µl of 2X buffer containing 0.4 mM of each dNTP and 2.4 mM MgSO_4_.

**Table 1:** RT-PCR primers information

mRNA	*GenBank* access	Primer pair sequence (5′ → 3′)	PCR Product (bp)	Annealing Temp (°C)
Na_V_β1	NM_001037	F: AGAAGGGCACTGAGGAGTTT R: GCAGCGATCTTCTTGTAGCA	379	60
Na_V_β2	NM_004588	F: GCCCACCCGACTAACATCTC R: ATGCGGAACTGGAGGAACA	285	62
Na_V_β3	NM_018400	F: CTGGCTTCTCTCGTGCTTAT R: TCAAACTCCCGGGACACATT	353	60
Na_V_β4	NM_174934	F: CACGCCACCATCTTCCTCCAA R: TGCAGCTGCTCAGCCCGAAG	284	65
β-actin	NM_001101	F: GCTCGTCGTCGACAACGGCTC R: CAAACATGATCTGGGTCATCTTCTC	353	60

*F* forward primer, *R* reverse primer

### Real-time PCR (qPCR)

Total RNA was extracted using the RNeasy Mini Kit (Qiagen; Hilden, Germany), then reverse-transcribed with the High Capacity cDNA Reverse Transcription kit (Applied Biosystems; Foster City, CA) according to the manufacturer’s instructions using 2 µg of total RNA in a final volume of 20 µl. Real-time PCR was carried out in a Rotor-Gene Q (Qiagen) using Custom TaqMan Gene Expression Assays (Applied Biosystems) as described before [14]. Briefly, 100 ng of cDNA, 0.4 µl of the TaqMan assay (Table 2) and 5 µl of TaqMan Universal PCR Master Mix (Applied Biosystems) were mixed in a final reaction volume of 10 µl for each qPCR reaction. At least three independent experiments were done, and each assay was performed in triplicate. The results were analyzed by the 2^−ΔΔCt^ method [31] using HPRT1 expression as the normalizing gene control and results are shown as relative expression values of Na_V_β1 in HeLa cells.

**Table 2:** qPCR primers information

mRNA	*GenBank* access	Primer pair sequence (5′ → 3′)	PCR product (bp)
Na_V_β1	NM_001037	F: GGAGGATGAGCGCTTCGA R: CAGATCCTGCAGGTCTTTGGT P: CCCCGGCTGCCATT	70
Na_V_β2	NM_004588	F: TGCAGCCGGAGGATGAG R: GAGGACCTGCAGATGGATCTTG P: CCCCTGACCGCCACCG	92
Na_V_β3	NM_018400	F: CGCCAGCCCCAGAAGAT R: CACAGGGAAGCAGACACTGA P: TTTCCCCTGGCTTCTC	90
Na_V_β4	NM_174934	F: AAGAAGTGGACAACACAGTGACA R: TGAGTTTCTTGATCAGCAGGATGAG P: ACCCCGCCCACGACAG	93

*F* forward primer, *R* reverse primer, *P* TaqMan probe

### Western blot

Total protein from native or transiently transfected CeCa cells was extracted 24, 48, 72 and 96 h post-transfection (with cDNA or siRNAs, for overexpression or inhibition of the Na_V_β expression respectively) using RIPA buffer (25 mM Tris–HCl, pH 7.4; 150 mM NaCl; 1% IGEPAL; 1% Sodium deoxycholate, and 1% SDS) supplemented with complete EDTA-free protease inhibitors (Roche, Switzerland), and quantified by Bradford assay. Equal amounts of protein (100 µg) were subjected to SDS-PAGE, transferred onto a polyvinylidene difluoride membrane (Millipore, Billerica MA) and probed overnight with the following primary antibodies: rabbit anti-Na_V_β1 (1:3000; LifeSpan BioSciences Inc.; Seattle, WA); rabbit anti-Na_V_β2 (1:1000; LifeSpan BioSciences Inc.); rabbit anti-Na_V_β3 (1:5000; Abcam; Cambridge, UK), rabbit anti-Na_V_β4 (1:3000; Novus Biologicals; Littleton, CO) and a homemade mouse anti-β-actin antibody (1:1000) used as a loading control. Blots were subsequently probed with an anti-rabbit or an anti-mouse (as the case may be) secondary antibody conjugated with horseradish peroxidase (1:10,000; Santa Cruz Biotechnology; Dallas, TX) for 1 h at room temperature and visualized using the SuperSignal West Pico chemiluminescent substrate (Thermo Fisher Scientific). Signal intensity of immunoblots was calculated with ImageJ software.

### Proliferation assays

Cell proliferation was determined using the colorimetric 3-[4,5-dimethylthiazol-2-yl]-2,5-diphenyltetrazolium bromide (MTT) assay. Briefly, cells were plated in 48-well microplates at an initial density of 2 × 10^3^ cells per well. To measure cell proliferation at 24, 48, 72 and 96 h after seeding, 10 µl of MTT solution (5 mg/ml) were added in each well after replacing the culture medium with 200 µl of fresh culture medium. The cells were incubated at 37 °C, and 3 h later, cell culture and MTT solution were removed and 150 µl of dimethyl sulfoxide was added into the plate to dissolve formazan crystals. The absorbance at a wavelength of 562 nm was recorded using an Eppendorf AG 22331 Biophotometer (Eppendorf; Hamburg, Germany). For analyzing the role of Na_V_βs on proliferation, after overnight incubation, cells were transfected with the respective cDNA to overexpress or the specific siRNA to downregulate each β-subunit expression, and finally, MTT assay was performed 24, 48, 72 and 96 h after transfection.

### Cell cycle analysis by flow cytometry

The role of Na_V_β1 on cell cycle in SiHa cells was assessed by flow cytometry after staining the cells with propidium iodide (PI; Molecular Probes, Life Technologies; Grand Island, NY). Briefly, 2 × 10^5^ cells were seeded onto 12-well plates, incubated in normal conditions for 24 h, synchronized by serum-reduced conditions (0.2% FBS) for 48 h and then transfected with the specific cDNA or siRNA. After incubating for 48 h, cells were collected and fixed with ice-cold 70% ethanol, stored for at least 24 h at − 20 °C, treated with RNase and stained with PI for 1 h at 37 °C. For each condition, 2 × 10^4^ cells (singlets) were analyzed in an Accuri C6 cytometer (BD Biosciences; San Jose, CA), and the percentages of cells in each cell cycle phase were analyzed with FlowJo software (Tree Star; Ashland, OR) using the Dean-Jett-Fox algorithm by three independent tests for each condition. Appropriate gating was used to select the single cell population and used on all samples.

### In vitro migration and invasion assays

Migration and invasion assays were done using culture inserts with 8-µm pore size membranes covered or not with Matrigel (Corning Inc., Corning, NY) for invasion and migration respectively. The upper compartment was seeded with 5 × 10^4^ for migration and 1 × 10^5^ cells for invasion in DMEM supplemented with 5% FBS, and the lower compartment was filled with DMEM supplemented with 15% FBS as a chemoattractant. The number of cells migrating or invading (according to the circumstances) over 48 h at 37 °C was evaluated using the MTT assay described before. Results were compiled as the mean of at least three repeats, each time done by duplicate, and compared with control conditions.

### In silico immunohistochemistry (IHC) staining analysis

Expression of Na_V_β4 in CeCa and normal cervical tissues was examined using the Human Protein Atlas (http://www.proteinatlas.org/) [32, 33], a Swedish-based online tool for human protein expression analysis. Representative CeCa and normal tissues cores were chosen for illustrative purposes.

### Statistical analysis

All quantitative results are given as the mean ± S.D. Differences in means were tested with an unpaired two-tailed Student’s *t* test.

## Results

### Cervical cancer cell lines do not express plasma-membrane voltage-gated sodium currents

Our previous results showed the functional expression of Na_**V**_ channels in CeCa primary cultures and specifically demonstrated the relevance of the Na_**V**_1.6 channel in the invasiveness of cervical cancer [14, 30]. Thus, in order to further investigate the role of Na_**V**_ channels in the biology of CeCa, we intended to use CeCa cell lines for such experiments. However, we found that SiHa, CaSki and HeLa cell lines do not express functional Na_**V**_ channels at the plasma-membrane as determined by whole-cell patch-clamp experiments. (*n* = 30 cells total; see Additional file 2), even though we have previously shown that they do express the Na_**V**_1.6 mRNA and at least one isoform of the Na_**V**_1.6 protein [34].

### Endogenous expression of Na_V_βs in cervical cancer cell lines

**Fig. 1:**
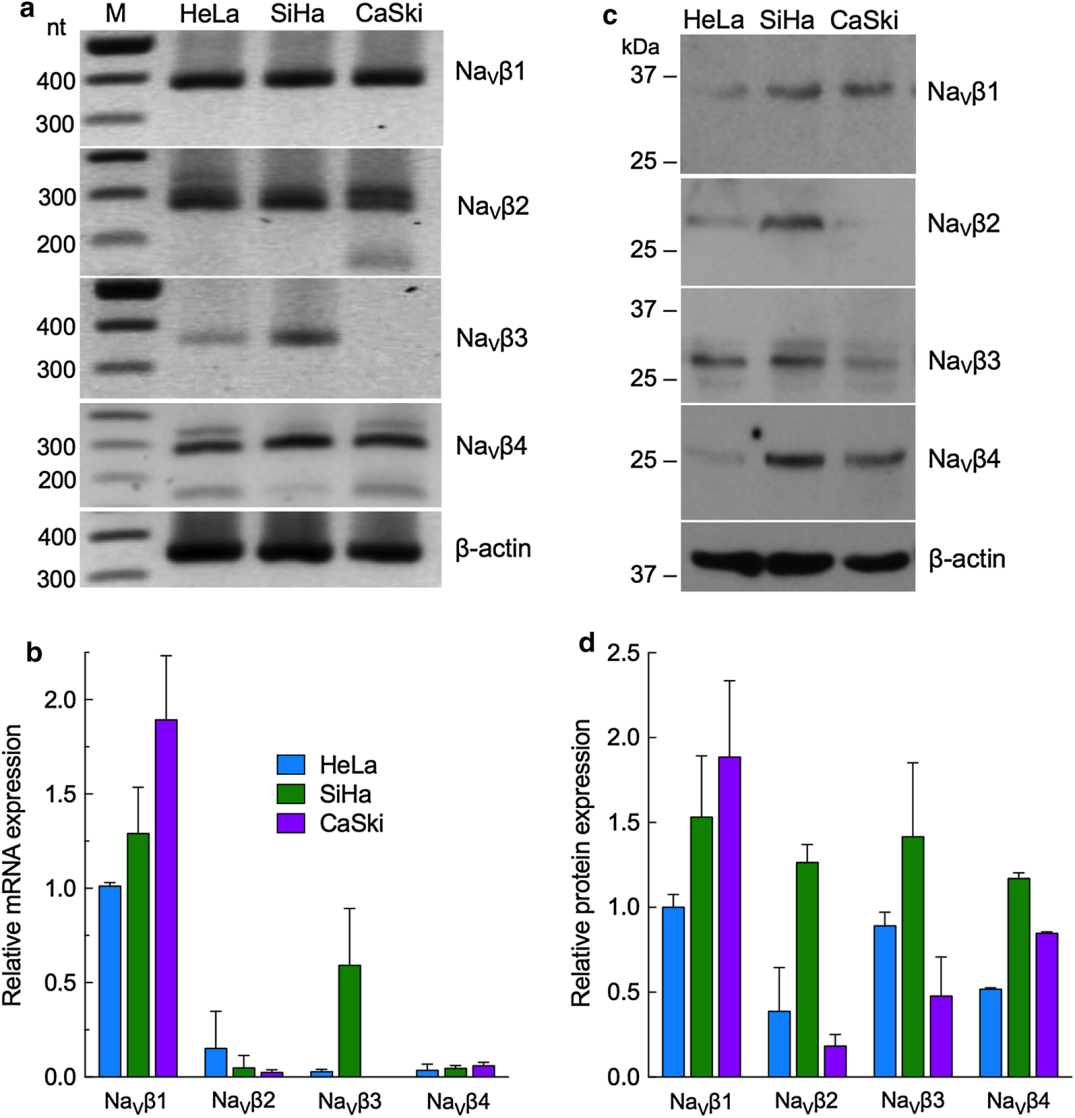
Expression of Na_V_βs in cervical cancer cell lines. **a** Total RNA isolated from CeCa cell lines was analyzed by conventional RT-PCR using specific primers for the indicated Na_V_β and β-actin as a loading control. Expected fragment sizes for each gene are shown on the right of each panel. M: molecular marker. **b** Quantitative differences in Na_V_βs mRNA expression of CeCa cells analyzed by qPCR, normalized with HPRT1 mRNA levels and relative to Na_V_β1 in HeLa. Each bar denotes mean ± S.D. of three independent experiments. **c** Western blot with 100 µg of total protein per lane from each of the cell lines indicated. Specific antibodies against each Na_V_β were used and β-actin antibody as a control for loading. **d** Quantified data for Na_V_βs protein expression normalized with β-actin expression for each cell line, and relative to Na_V_β1 expression in HeLa. Each bar denotes mean ± S.D. of three independent experiments

In previous studies, we described the role of Na_V_ α-subunits in the invasive potential of CeCa primary cultures. In this work, we wanted to analyze the possible role of Na_V_βs in different CeCa cellular processes. To that end, endogenous expression of Na_V_βs was first evaluated by RT-PCR using specific primers for each subunit. In most of cases, the mRNA was abundant as evidenced by the generation of the expected amplicon for each β-subunit (Fig. 1a), suggesting the very likely presence of these proteins. Along with the expected bands, additional PCR amplicons were observed when analyzing the expression of Na_V_β2 and Na_V_β4 by RT-PCR (Fig. 1a). The ≈ 150 bp DNA fragment observed in the Na_V_β4 line matches with a splice variant that has been already reported [23], that would produce a highly truncated protein (if translated) with an unknown functionality. The larger non-specific bands in Na_V_β2 and Na_V_β4 RT-PCR do not correspond to any reported variants of such isoforms, and we cannot rule out the possibility of a non-specific amplification with these primers. A more detailed study is required to analyze the Na_V_βs isoforms expressed in the CeCa cells and their potential function and/or contribution to the cancerous phenotype. Nevertheless, to carry out a more quantitative analysis of the Na_V_βs expression levels in the different CeCa cell lines we performed qPCR experiments. Results exhibited substantial differences in Na_V_βs mRNA expression, but in all cases, Na_V_β1 showed to be highly expressed, whereas Na_V_β3 displayed the lowest expression levels, except in SiHa cells (Fig. 1b). In addition, evaluation of protein expression by western blot for each Na_V_β indicates that the four Na_V_βs are expressed in the three CeCa cell lines (Fig. 1c). Interestingly, SiHa was the only cell line that express very similar levels of all four Na_V_βs (Fig. 1d). Also, SiHa and CaSki cells expressed significantly higher levels of Na_V_β1 and Na_V_β4 than HeLa cells. While Na_V_β3 levels were similar among the CeCa cell lines, and Na_V_β2 was the lees expressed except in SiHa cells (Fig. 1d). The western blot results suggest the absence of additional variants of Na_V_βs isoforms, as we only observed the protein of the expected size.

### Proliferation, migration and invasion of CeCa cell lines under basal conditions

**Fig. 2:**
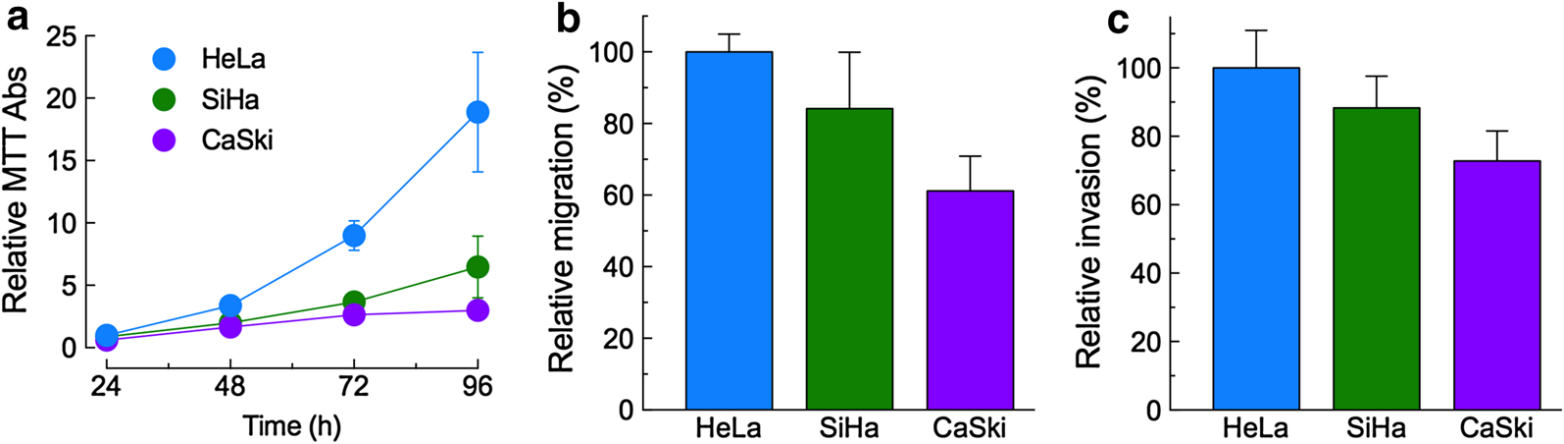
Proliferation, migration and invasion of CeCa cells. **a** Proliferation was evaluated by MTT assays 24, 48, 72 and 96 h after seeding the cells (2 × 10^3^ per well). Means ± S.D. of three independent experiments, each one done by triplicate. Migration (**b**) and invasion (**c**) of CeCa cells were assessed by Boyden transwell assays for 48 h. Results expressed as percentages of values obtained for HeLa cells. Means ± S.D. of three independent experiments, each done by duplicate. HeLa cells showed to be the most aggressive, as they proliferated, migrated and invaded the most

In order to evaluate the metastatic potential of each CeCa cell line, we analyzed three key metastatic behaviors: proliferation, migration and invasiveness in vitro. HeLa cells demonstrated to be the most aggressive cell line as these cells proliferated, migrated and invaded the most, followed by SiHa cells, and CaSki cells (Fig. 2).

### Increasing or decreasing the expression of Na_V_βs is not enough to promote the functional activity of α-subunits in the plasma membrane

First, to evaluate the siRNA transfection efficacy, we used the siGLO Green Transfection Indicator, a fluorescent dye attached to a scramble siRNA, and analyzed the cells 24 h later by epifluorescent microscopy. The results indicated that almost 100% of the cells were transfected with the siRNAs, as can be appreciated in Additional file 3. Thus, we followed the same conditions to transfect the specific siRNAs against each of the Na_V_βs.

It is well known that some of the Na_V_βs are implicated in the correct trafficking of the Na_V_ α-subunits to the plasma membrane, as has been shown with the heterologous in vitro expression of Na_V_β1 and Na_V_β2 in Xenopus oocytes [15, 35], and in vivo with Na_V_β2 knocked-out mice [36]. These evidences made us wonder if the sole overexpression or even the down regulation of the expression of Na_V_βs was enough to promote the functional expression of the pore-forming sodium channels in the plasma membrane and therefore the appearance of voltage-gated sodium currents. To address this question, HeLa and SiHa cells were analyzed by the whole-cell patch-clamp technique 24, 48, 72 and 96 h after transfection to increase or decrease the expression of each of the Na_V_βs. More than 130 cells were successfully investigated with this technique, but we did not find any voltage-gated sodium currents, suggesting that the regulation of the expression of Na_V_βs is not enough to promote the functional activity of Na_V_ α-subunits in the plasma membrane (see Additional file 2).

### Role of Na_V_βs on proliferation

**Fig. 3:**
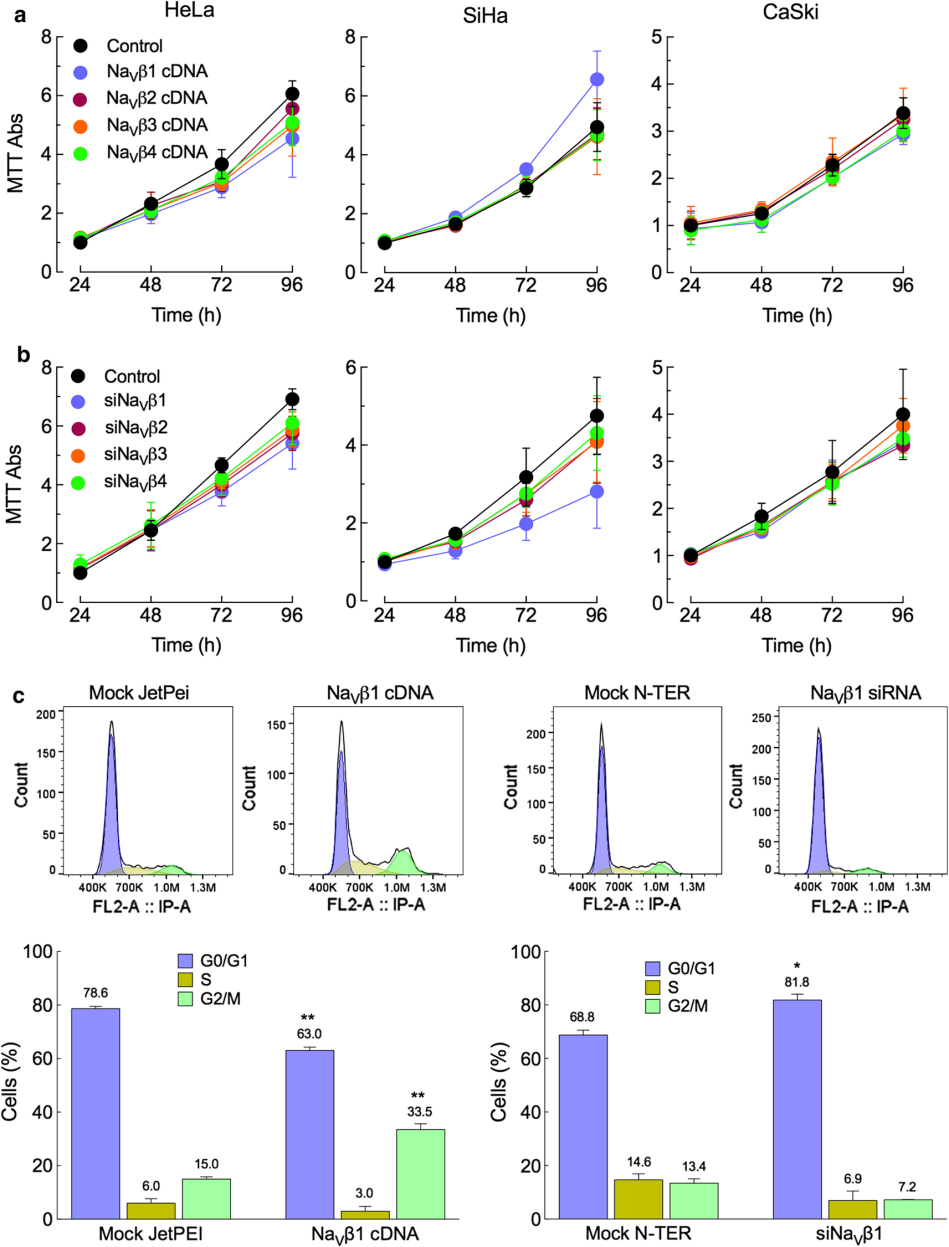
Effect of Na_V_βs on proliferation and cell-cycle of CeCa cells. Proliferation was evaluated by MTT assays 24, 48, 72 and 96 h post-transfection with plasmids (**a**) or with specific siRNAs (**b**) for the β-subunits. The results show the participation of Na_V_β1 on proliferation of SiHa cells, as it increases when the protein is overexpressed and decreases when it is downregulated. The other three Na_V_βs showed no effect on proliferation of SiHa cells, and none of the β-subunits were involved in proliferation of HeLa and CaSki cells. **c** Cell cycle analysis by flow cytometry in SiHa cells performed 48 h after transfection of synchronized cells with Na_V_β1 cDNA or with the specific siRNA against this subunit. Cells were fixed with ice-cold 70% ethanol, stored for at least 24 h at − 20 °C, treated with RNase and stained with PI for 1 h at 37 °C. For each condition, 2 × 10^4^ cells (singlets) were analyzed. Representative histograms of the control mock conditions and the transfected cells (upper panels) of three independent experiments summarized in the bar graphs (bottom panels) as means ± S.D. of the relative percentage of cells in each cell cycle phase. Significance: *P < 0.05, **P < 0.01

Next, we investigated whether the expression of Na_V_βs had an effect on cell proliferation. This was evaluated in CeCa cell lines 24, 48, 72 and 96 h post-transfection with the cDNA or with the specific siRNA for each Na_V_β subunit to increase or to reduce the expression respectively. Only Na_V_β1 had an effect on proliferation of SiHa cells: the overexpression of Na_V_β1 increased cell proliferation, whereas its downregulation decreased cell duplication (Fig. 3a; center panels). This is similar to what was found with the breast cancer cell line MDA-MB-231, where the overexpression of Na_V_β1 in these cells led to the development of larger tumors in mice [28]. To corroborate this observation, we decided to perform a cell cycle assay by flow cytometry. First, for standardization purposes, SiHa cells were arrested at the G0/G1 phase by incubation in serum-reduced conditions for 24 and 48 h. To estimate the proportion of cells in different phases of the cell cycle, cells were stained with PI and analyzed by flow cytometry. Results show a consistent arrest of cells when treated for 48 h, as the proportion of the cells in G0/G1 phase increased significantly whereas the percentage of cells in G2/M phase was smaller compared to control (normal medium) conditions (see Additional file 4). Once synchronized cells were transfected with either cDNA or siRNA for Na_V_β1 subunit under the experimental conditions indicated above. Results show a significant increase in the proportion of cells in G2/M phase when overexpressing Na_V_β1 vs the control condition (JetPEI mock control), as well as a significant decrease in the proportion of cells in G0/G1 phase. In the same sense, the downregulation of the expression of Na_V_β1 generated a significantly larger proportion of cells in G0/G1 phase, with no significant changes in the proportion of cells in G2/M phase compared with the N-TER mock control (Fig. 3b). These results suggest a positive role of Na_V_β1 in the proliferation of SiHa cells, but not in HeLa or CaSki cells.

### Role of Na_V_βs on migration and invasion

**Fig. 4:**
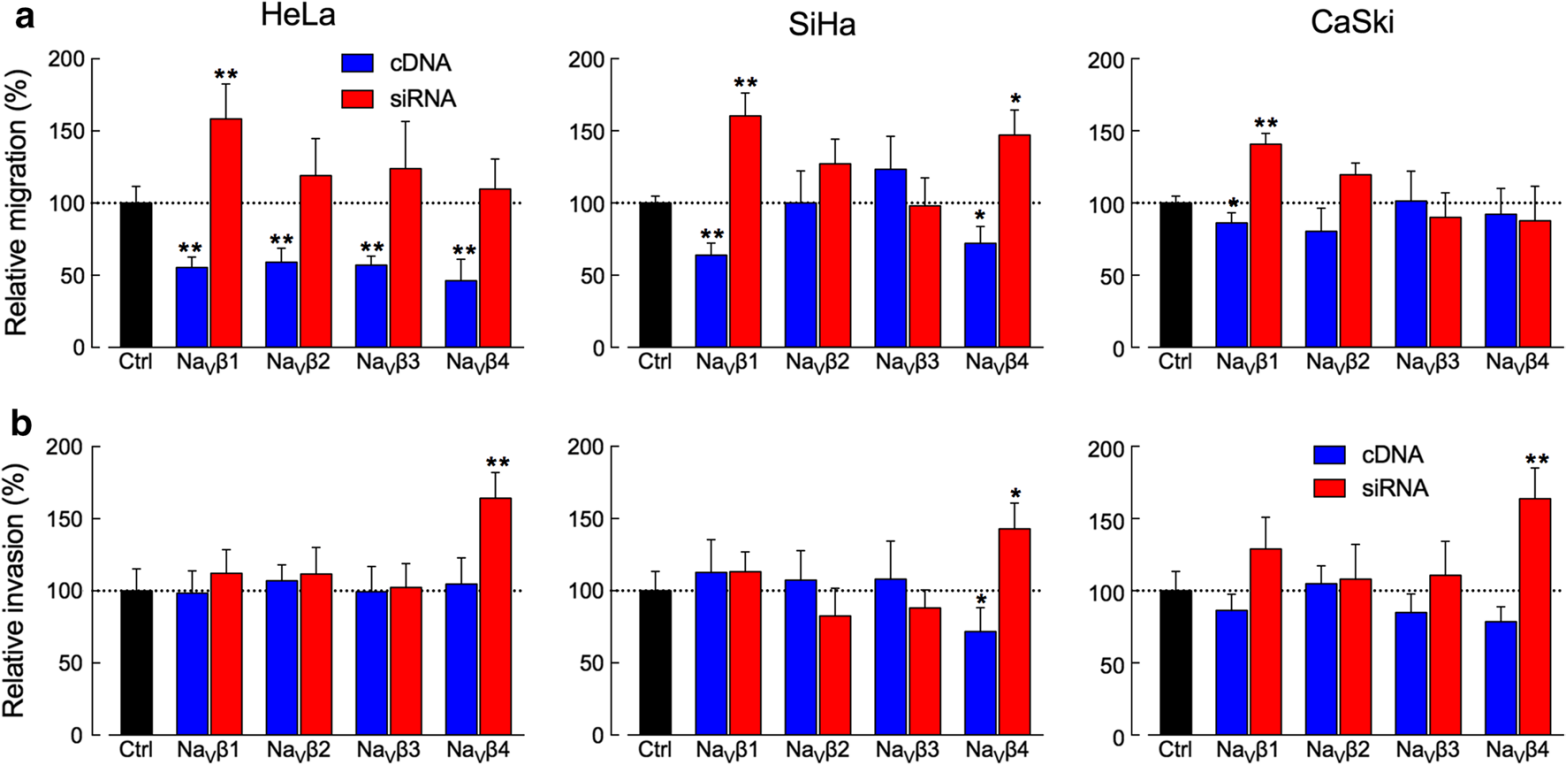
Effect of Na_V_βs on migration and invasiveness of CeCa cells. Migration (**a**) and invasion (**b**) experiments were performed with HeLa, SiHa and CaSki cells in Boyden transwell chambers for 48 h. Bars indicate mean ± S.D. of at least three independent experiments, each performed in duplicate and presented as a percentage of control conditions. Overexpression of Na_V_βs in HeLa cells induced a reduction in approximately 50% in cellular migration, while the downregulation of Na_V_β1 by siRNAs increased it in 58%. Na_V_β1 is also involved in SiHa and CaSki migration. On the other hand, downregulation of Na_V_β4 with siRNAs increased cellular invasiveness in the three cell lines. Significance: *P < 0.05, **P < 0.01

The role of Na_V_βs on in vitro migration and invasion of CeCa cell lines was evaluated 48 h post-transfection with the cDNA or with the siRNA for each β-subunit to overexpress or to downregulate the expression of Na_V_βs respectively. Migration results show that overexpression of Na_V_βs in HeLa cells induces a significant reduction in migration capability (around 50% in all cases), regardless of the type of Na_V_β, accompanied by a tendency to increase the invasive capability when the Na_V_βs are downregulated, being the effect of the treatment with siRNAs against Na_V_β1 the most substantial (a 58% increment). In SiHa cells, a similar result was found with Na_V_β1 and Na_V_β4; a significant reduction on the migration capability (36% and 28% respectively) when they are overexpressed, and an increment (60% and 47% respectively) when they are downregulated, while the manipulation of the expression of Na_V_β2 and Na_V_β3 did not alter the migration of these cells. Finally, in CaSki cells, only Na_V_β1 showed to have a role on cellular migration: the overexpression of this subunit decreased the migration by 14%, whereas the transfection with siRNAs increased it by 41% (Fig. 4a). It is worth of notice that only the effect of Na_V_β1 (negative regulation) in migration remains constant and statistically significant in the three cell lines, suggesting a potential role of Na_V_β1 in CeCa migration capability.

With regards to the in vitro invasion, results show that the downregulation of Na_V_β4 increased the invasiveness of the three cell lines: 64% in HeLa, 43% in SiHa and 63% in CaSki cells. Accordingly, the overexpression of this protein induced a reduction on the invasiveness of SiHa and CaSki cells by 28% and 21% respectively, suggesting a potential role of Na_V_β4 as an invasion suppressor. The rest of the Na_V_βs did not significantly modified the invasion capacity of the CeCa cells (Fig. 4b).

Given all these results, we decided to corroborate whereas the diminished expression of Na_V_β4 was associated with the presence of CeCa vs normal cervix tissues. For that purpose, we analyzed in silico the expression Na_V_β4 in CeCa biopsies and normal cervical tissues from the Human Protein Atlas. Although the database only has a small number of IHC normal cervix and CeCa biopsies stained for Na_V_β4 (n = 3 for normal cervix, and n = 12 for cervical cancer), the relationship between the expression of the protein and the presence or absence of cervical cancer is evident: the staining results indicate that three out of three normal cervix tissues have moderate Na_V_β4 expression, while in the CeCa biopsies no signal of Na_V_β4 was detected in twelve out of twelve cases (see Additional file 5). Taken together, these results strongly suggest that Na_V_β4 expression is downregulated in CeCa, leading to an increase in the invasive capacity of the cells, and thus, the metastatic potential.

## Discussion

For many years, Na_V_βs were considered only as “auxiliary” Na_V_ subunits commissioned of regulating the α-subunit kinetics and cellular localization. Nowadays plenty of evidence shows the multifunctionality of these proteins, participating in a wide variety of cellular processes like cell adhesion, cytoskeleton remodeling, filopodia and invadopodia formation, transcriptional regulation, cell cycle regulation and even apoptosis [21, 29].

For the present work, we chose three different CeCa cell lines in order to cover a wide range of CeCa cases, and to analyze the potential role of Na_V_βs in the malignant behavior of this disease. SiHa and CaSki cells are HPV-16 positive (the most frequently detected viral type in CeCa, found in approximately 57% of cases) whereas HeLa cells arise from a HPV-18 positive tumor (the second most frequent virus; 16% of cases) [37]. Furthermore, HeLa and SiHa cells derived from a primary tumor, while CaSki cells were taken from a metastatic tumor. In addition, the patients’ ethnicity and the cellular origins of the three cell lines are different [38–40].

Here we demonstrated the presence (expression) of Na_V_βs in the three different CeCa cell lines (Fig. 1), even in the absence of functional Na_V_ α-subunit expression in the plasma membrane (evidenced by whole-cell patch-clamp recordings, see Additional file 2), suggesting that their role in cancer cell biology is independent of the pore-forming Na_V_ α-subunit.

When we evaluated the aggressiveness of each cell line, by analyzing the basal proliferation, migration and invasiveness in vitro, HeLa cells demonstrated to be the most aggressive cell line, as these cells proliferated, migrated and invaded the most, followed by SiHa cells. CaSki cells were the less proliferative, and they migrated and invaded the less (Fig. 2). As far as we are concerned, this is the first report about the differences in the metastatic behavior among these CeCa cell lines.

We have previously reported the role of Na_V_1.6 (the most overexpressed Na_V_ α-subunit in CeCa biopsies) in the invasiveness potential of CeCa primary cultures [14]. In agreement with our recent findings [34], in the present work we did not find voltage-gated sodium currents in any of the CeCa cell lines studied with the patch-clamp technique. This could be due to at least two possibilities: either the sodium channels are being correctly synthetized but are not reaching the plasma membrane for some unknown reason; or these cells are expressing a non-functional splicing variant. In this regard, it has been reported that Na_V_βs participate in the appropriate traffic and localization of the Na_V_ α-subunits in the plasma membrane [15, 35, 36]. Moreover, it has been shown that Na_V_β2 can be cleaved by secretases, generating small intracellular peptides capable of reaching the cell nucleus and promoting the transcriptional upregulation of genes including SCN1A, which encodes for Na_V_1.1 [41, 42]. Hence, we thought that maybe by modifying the expression of these proteins we could promote the functional localization of conducting Na_V_ channels in the plasma membrane, however our results suggest this is not the case, as the manipulation of the expression of the Na_V_βs in HeLa and SiHa cells was not enough to promote the appearance of voltage-dependent sodium currents (see Additional file 2). Yet, there are many other molecules such as hormones and growth factors that could be involved in the correct trafficking of the channel. In fact, we have examined the possibility that Na_V_s functional expression in CeCa cell lines could be regulated by β-estradiol or EGF, but we did not find sodium currents under any of the experimental conditions tested (data not shown). Another likely explanation could be related to the oxygen conditions in which the cell lines are cultured. It has been reported that hypoxia can change both the expression and activity of Na_V_s. Tumor cells usually experience severe hypoxia, which has been correlated with a more robust expression and activity of Na_V_s [43, 44]. On the contrary, CeCa cell lines have been cultured in normoxic tissue culture conditions, which might have modified the Na_V_s expression and/or trafficking.

It is also possible that CeCa cell lines are expressing a non-conducting channel with a different and unknown function and/or localization in the cell. It remains to be investigated whether Na_V_ α-subunits are expressed in intracellular compartments, as well as their possible role in invasion. The existence of functional intracellular sodium channels has been demonstrated in macrophages where the activity of this sodium channel contributes to cellular invasion through a mechanism involving sodium ions release from cationic intracellular stores, followed by a subsequent mitochondrial calcium release mediated by the Na^+^/Ca^2+^ exchanger, which in turn facilitates cytoskeletal remodeling and invadopodia formation [12]. In fact, recent data from our group suggests the presence of intracellular Na_V_1.6 channels in CeCa cell lines [34]. However, more experiments are needed to fully elucidate this discrepancy between Na_V_s functional expression in CeCa primary cultures vs cell lines.

Regarding to the effect of Na_V_βs on cell proliferation, we found that only Na_V_β1 had an effect on this process and only in SiHa cells: the overexpression of Na_V_β1 increased cell proliferation, whereas the downregulation of the expression of this protein decreased it (Fig. 3a). This is in agreement with observations reported by Nelson et al. [28] demonstrating that Na_V_β1 enhances breast tumor growth and metastasis in vivo, by increasing cell proliferation and reducing apoptosis. The fact that we only observed this effect in one out of three CeCa cell lines shows that it is a particularity of the cell line (SiHa) rather than a general role of Na_V_β1 on proliferation in cervical cancer.

On the other hand, it has been suggested that Na_V_β3 mediates a p53-dependent apoptotic pathway [26]. In breast cancer cells, for example, this subunit is totally absent, although it is normally expressed in non-cancerous breast cells (Sanchez-Sandoval et al., unpublished data). However, the overexpression or down regulation of this subunit did not alter the proliferation rate of the CeCa cell lines analyzed here, suggesting a lack of the pro-apoptotic activity of Na_V_β3 in these cells. This might be related to the p53 protein status in CeCa cell lines. It has been reported that the association between the E6 protein (one of the two main HPV oncogenes) with p53 leads to the specific ubiquitination and degradation of p53 protein [45], therefore inactivating any pro-apoptotic effect due to the Na_V_β3 expression in basal conditions. This is one of the several ways the HPV genome secures the growing of the tumor. However, the intermediate steps that Na_V_β3 must undergo to promote apoptosis is still unknown, as well as why Na_V_β3 had no pro-apoptotic effect in CeCa cells when overexpressed. A possible explanation could be the existence of a positive (and necessary) feedback regulation between p53 (or one of its multiple target genes) and Na_V_β3, leading to a lack of pro-apoptotic activity of Na_V_β3 in the absence of functional p53.

On the contrary, our migration experiments show a clear participation (negative regulation) of Na_V_β1 on the ability of CeCa cells to perform transwell migration across of an 8 µm-pore membrane. When we induced the overexpression of this subunit, the migration rate decreased in the three CeCa cell lines, being HeLa cells the most affected (almost 50% inhibition in cell migration). Correspondingly, the inhibition of Na_V_β1 expression by siRNAs led to significant increments in cell migration of HeLa, SiHa and CaSki cells (Fig. 4a). Thus, Na_V_β1 could be acting as a cell adhesion molecule, promoting the cell–cell adhesion and therefore making it difficult to move across the membrane. It is also worth of notice that, among the three cell lines, HeLa have the lowest Na_V_β1 expression compared to the other two CeCa cell lines (Fig. 1), and they also have the greatest migration capacity. In the same sense, CaSki cells are the ones with the bigger expression of Na_V_β1, and the lowest migration behavior (Fig. 2b).

A study made on breast cancer cell lines revealed that the overexpression of Na_V_β1 in highly metastatic MDA-MB-231 cells increased cell–cell adhesion and decreased in vitro migration, consistent with the proposed role of Na_V_β1 as a cell adhesion molecule [27]. However, the *trans*-homophilic interactions through Na_V_β1 can also mediate the process outgrowth in vivo, as in neurons, generating an elongated morphology and therefore potentiating metastasis, as has been reported for the same breast cancer cells but using mouse models instead of transwell experiments [28]. Something similar could be happening with CeCa cell lines. Thus, the persistent contribution of Na_V_β1 in the three CeCa cell lines migration is indicative of its role as a negative regulator of the in vitro migration, regardless the HPV type present in the CeCa cell line. Although it remains to be investigated whether this behavior also occurs in vivo.

With respect to the contribution of Na_V_βs in invasiveness, our results show that downregulation of Na_V_β4 expression resulted in a significant increase in the in vitro invasiveness of the three cell lines studied here (Fig. 4b), suggesting the role of this subunit as a metastasis suppressor protein. These observations agree with the in silico analysis of Na_V_β4 expression performed with the Human Protein Atlas data: normal cervix biopsies show moderated Na_V_β4 expression, while in CeCa biopsies it seems to be absent (see Additional file 5). Taken together, these results strongly suggest that Na_V_β4 expression is downregulated in CeCa, which in turn increases the invasive capacity of the cells. In accordance with this interpretation, out of the three CeCa cell lines studied in the present work, the HeLa cell line showed the lowest expression of Na_V_β4 and the highest index of cell invasion.

In breast cancer cell lines, it has been found that increasing Na_V_β1 expression promotes the overexpression of Na_V_1.5, the main Na_V_ α-subunit involved in the invasiveness of this type of cancer [27]. However, according to our results, this is not the case for CeCa cell lines, as the overexpression of Na_V_β1 or Na_V_β4 did not promote the expression of conducting Na_V_1.6 channels in the plasma membrane, the principal Na_V_ α-subunit involved in the invasive potential of these cells. Thus, we conclude that the pathways used by these proteins to induce cell migration and invasion, are independent of the activity of Na_V_1.6 or other Na_V_ α-subunits in the plasma membrane of CeCa cell lines.

Other groups have also reported the downregulation of Na_V_β4 expression in cancer vs normal tissue. This is the case of prostate cancer [23], papillary thyroid cancer [46], and breast cancer [29]. The latter study, performed by the group of Dr. Sebastien Roger at the University of Tours, reported solid evidence about the possible mechanistic pathway through which Na_V_β4 prevents metastasis in cancer cells. They showed an increased activity of the small GTPase RhoA (crucial for cytoskeleton remodeling during migration and invasion) when Na_V_β4 was silenced in breast cancer MDA-MB-231 cells. This effect was independent of Na_V_1.5 channel activity, the main Na_V_ α-subunit implicated in BCa invasiveness. A similar mechanism might be taking place in CeCa cells: a downregulation of the Na_V_β4 subunit that leads to an increased activity of RhoA and therefore, a more robust invasive potential, which is independent of the Na_V_1.6 expression in the plasma membrane. However, we speculate that additional cellular mechanisms might be involved in this phenomenon, as the effects of Na_V_β4 in cellular migration were not significant in our study, meaning that the sole change on cellular motility cannot explain the dramatic increase on cellular invasiveness when Na_V_β4 is downregulated. A more recent study showed that Na_V_β4 is downregulated in papillary thyroid cancer (PTC) compared with normal thyroid tissues. More interestingly, the authors also found that preserved Na_V_β4 expression might independently predict favorable recurrence-free survival in classical PTC [46]. Altogether, these data reinforce the notion of using Na_V_β4 as a biomarker for cancer metastasis and a potential new therapeutic target for the treatment of cervical cancer.

## Conclusions

In conclusion, our results show that even though cervical cancer cell lines do not generate Na_V_ currents, they do express Na_V_β subunits, which could play a role in proliferation (Na_V_β1 only in SiHa cells), migration (Na_V_β1 in the three cell lines), and invasion (Na_V_β4 in the three cell lines studied). The results support the recent knowledge about Na_V_βs as multifunctional proteins involved in cell processes like ion channel regulation, cell adhesion and motility, and even in metastatic cell behaviors. Finally, the evidence presented here contributed to propose Na_V_β1 and Na_V_β4 as prognostic markers for cervical cancer tumor progression.

## Additional files

**Additional file 1.** Evaluation of Na_V_βs expression after transfecting HeLa cells with siRNAs or cDNAs. Real-time PCR and western blot data of Na_V_βs expression in HeLa cells after transfection with corresponding cDNAs or siRNAs.

**Additional file 2.** Analysis of the expression of voltage-gated sodium currents in CeCa cells. Whole-cell patch-clamp experiments performed in CeCa cells to investigate the presence of functional Na_V_ α-subunits in the plasma membrane.

**Additional file 3.** Efficiency of siRNA transfection in CeCa cells. Micrographs of CeCa cells showing the efficiency of siRNA transfection using a fluorescent transfection indicator.

**Additional file 4.** Synchronization of SiHa cells. Flow cytometry results for synchronization of SiHa cells.

**Additional file 5.** Analysis of Na_V_β4 expression in CeCa biopsies and normal cervical tissue. Representative immunohistochemical images of cervical cancer biopsies and normal cervix tissues showing the absence of Na_V_β4 in CeCa biopsies and the moderate expression of the protein in normal cervix.

## Abbreviations

BCa: breast cancer
CAMs: cell adhesion molecules
CeCa: cervical cancer
DMEM: Dulbecco’s Modified Eagle’s Medium
FBS: fetal bovine serum
Na_V_: voltage-gated sodium channels
Na_V_βs: voltage-gated sodium channels β-subunits
Na_V_1.6: voltage-gated sodium channel coded by the *SCN8A* α-subunit
PI: propidium iodide
PTC: papillary thyroid cancer
qPCR: real-time polymerase chain reaction
RT-PCR: reverse transcription polymerase chain reaction
