# Supplementary material for: Contribution of voltage-gated sodium channel β-subunits to cervical cancer cells metastatic behavior
Source: Cancer Cell Int. 2019 Feb 15;19:35. doi: 10.1186/s12935-019-0757-6 (PMC6377746; doi:10.1186/s12935-019-0757-6)
Supplement: Supplementary file 1 — Additional file 1. Evaluation of NaVβs expression after transfecting HeLa cells with siRNAs or cDNAs. Real-time PCR and western blot data of NaVβs expression in HeLa cells after transfection with corresponding cDNAs or siRNAs. [file 12935_2019_757_MOESM1_ESM.pdf]

**Additional file 1.**

# Contribution of voltage-gated sodium channel β-subunits to cervical cancer cells metastatic behavior

**Ana Laura Sanchez-Sandoval, Juan Carlos Gomora**

Departamento de Neuropatología Molecular, División de Neurociencias, Instituto de Fisiología Celular, Universidad Nacional Autónoma de México. Ciudad de México, 04510, México.

*Corresponding author: jgomora@ifc.unam.mx

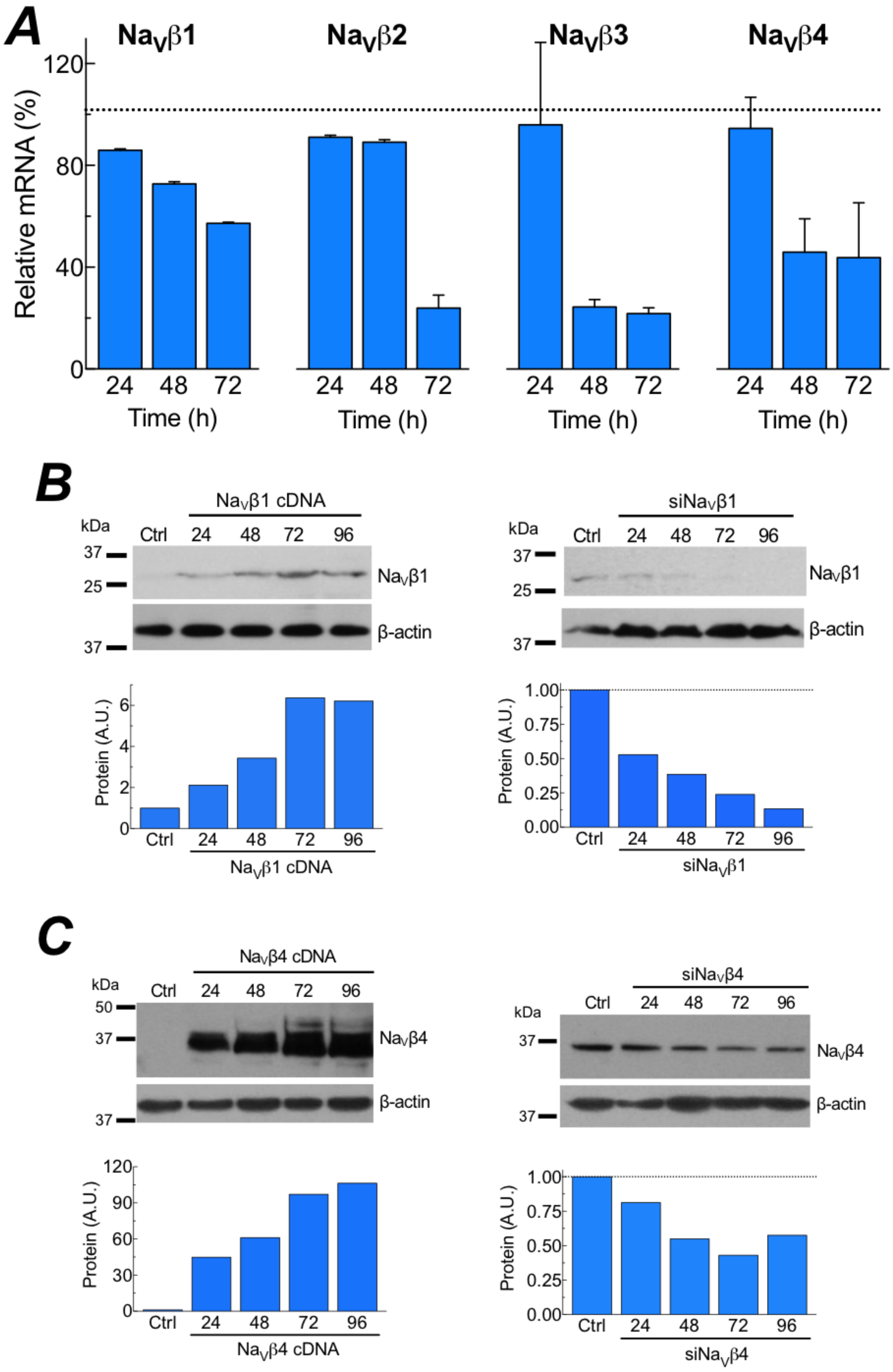
A
B
C
$Na_V\beta1$
$Na_V\beta2$
$Na_V\beta3$
$Na_V\beta4$
Relative mRNA (%)
Time (h)
$Na_V\beta1$ cDNA
$siNa_V\beta1$
$Na_V\beta4$ cDNA
$siNa_V\beta4$
kDa
Ctrl
β-actin
Protein (A.U.)

**Additional file 1. Evaluation of $Na_V\beta$s expression after transfecting HeLa cells with siRNAs or cDNAs.** ***A***. Real-Time PCR analysis of $Na_V\beta$s mRNA expression levels for HeLa cells 24, 48 and 72 h after transfection with 100 nM of the specific siRNAs for each subunit. Data was normalized with HPRT1 mRNA levels, and relative to each β-subunit mRNA expression in control (mock) conditions (100%, dotted line). Bars denotes mean ± S.D. ***B***, ***C***. Western blot analysis of $Na_V\beta1$ and $Na_V\beta4$ expression in transfected HeLa cells. Protein expression was assessed by using specific antibodies against $Na_V\beta1$ (***B***) or $Na_V\beta4$ (***C***) at 24, 48, 72 and 96 h post-transfection with the respective plasmid to overexpress (left panels) or with siRNAs (right panels) to deplete them. β-actin detection was used as a control for loading. Bar graphs show quantified data for $Na_V\beta$s protein expression normalized with β-actin expression for each condition and relative to control cells.
