# Supplementary material for: Contribution of voltage-gated sodium channel β-subunits to cervical cancer cells metastatic behavior
Source: Cancer Cell Int. 2019 Feb 15;19:35. doi: 10.1186/s12935-019-0757-6 (PMC6377746; doi:10.1186/s12935-019-0757-6)
Supplement: Supplementary file 2 — Additional file 2. Analysis of the expression of voltage-gated sodium currents in CeCa cells. Whole-cell patch-clamp experiments performed in CeCa cells to investigate the presence of functional NaV α-subunits in the plasma membrane. [file 12935_2019_757_MOESM2_ESM.pdf]

Additional file 2.

Contribution of voltage-gated sodium channel β-subunits to cervical cancer cells metastatic behavior

Ana Laura Sanchez-Sandoval, Juan Carlos Gomora*

Departamento de Neuropatología Molecular, División de Neurociencias, Instituto de Fisiología Celular, Universidad Nacional Autónoma de México. Ciudad de México, 04510, México.

*Corresponding author: jgomora@ifc.unam.mx

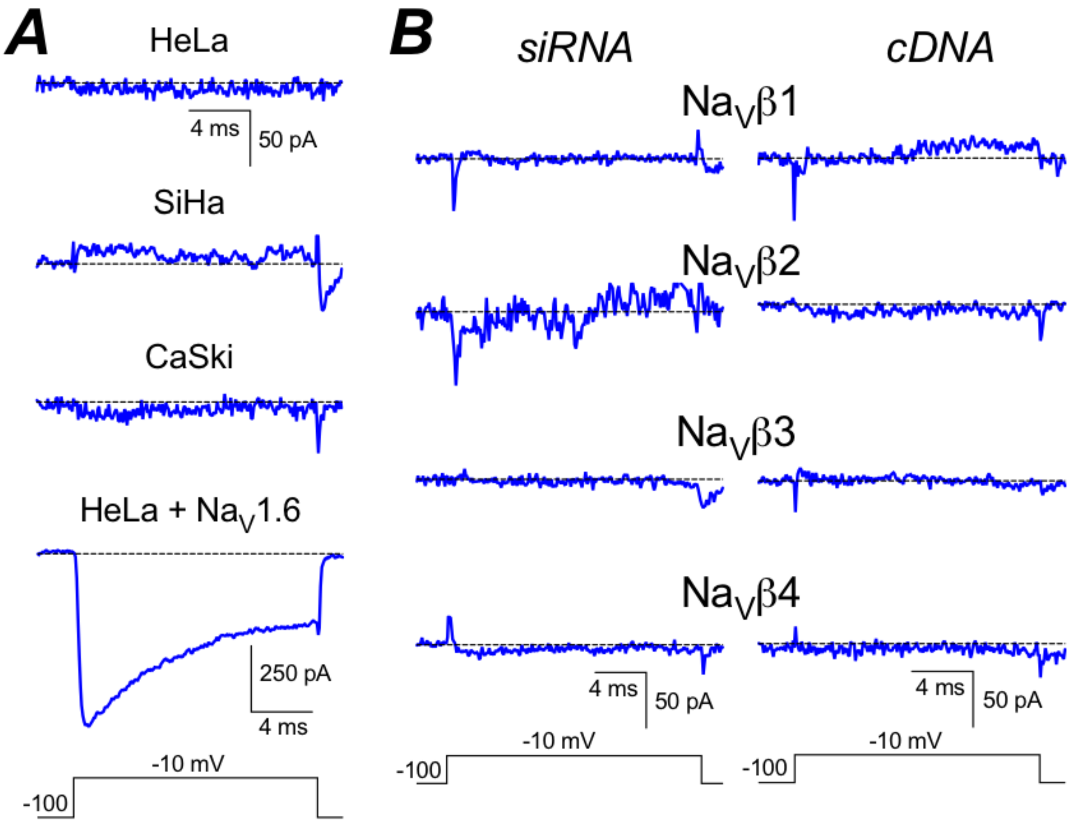

Additional file 2. Analysis of the expression of voltage-gated sodium currents in CeCa cells. Whole-cell patch-clamp experiments were done to investigate the presence of functional Na_V α-subunits in the plasma membrane in CeCa cells. ***A***. None of the three CeCa cell lines showed the presence of voltage-gated sodium currents in basal conditions (n = 30 in total), whereas prominent Na_V currents were recorded in HeLa cells transiently transfected with $Na_V1.6$, used as a positive control (bottom recording). ***B***. HeLa cells transfected with siRNAs (left column) or the cDNA (right column) to increase or decrease the expression of each of the $Na_V\beta$s, but no Na_V current was found, suggesting that the regulation of the expression of $Na_V\beta$s is not enough to promote the functional activity of Na_V α-subunits in the plasma membrane of CeCa cells. In all cases, currents were evoked at -10 mV from a holding potential of -100 mV.
