# Supplementary material for: Contribution of voltage-gated sodium channel β-subunits to cervical cancer cells metastatic behavior
Source: Cancer Cell Int. 2019 Feb 15;19:35. doi: 10.1186/s12935-019-0757-6 (PMC6377746; doi:10.1186/s12935-019-0757-6)
Supplement: Supplementary file 3 — Additional file 3. Efficiency of siRNA transfection in CeCa cells. Micrographs of CeCa cells showing the efficiency of siRNA transfection using a fluorescent transfection indicator. [file 12935_2019_757_MOESM3_ESM.pdf]

Additional file 3.

Contribution of voltage-gated sodium channel β-subunits to cervical cancer cells metastatic behavior

Ana Laura Sanchez-Sandoval, Juan Carlos Gomora*

Departamento de Neuropatología Molecular, División de Neurociencias, Instituto de Fisiología Celular, Universidad Nacional Autónoma de México. Ciudad de México, 04510, México.

*Corresponding author: jgomora@ifc.unam.mx

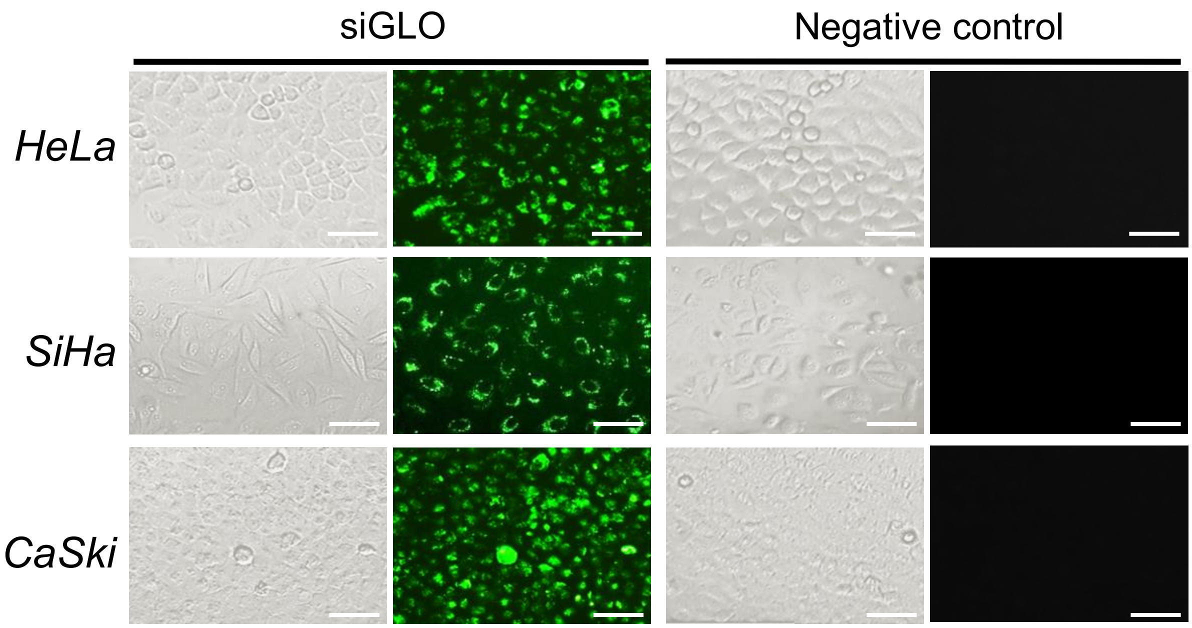

Additional file 3. Efficiency of siRNA transfection in CeCa cells. Representative digital pictures of HeLa, SiHa and CaSki cells transfected with 50 nM of the siGLO Green Transfection Indicator (left panels). Pictures were taken 24 h after transfection. Results showed high transfection efficiency in the three cell lines. Non-transfected cells (negative control; right panels) showed no fluorescence, as expected. Scale bar: 40 µm.
