# Supplementary material for: Contribution of voltage-gated sodium channel β-subunits to cervical cancer cells metastatic behavior
Source: Cancer Cell Int. 2019 Feb 15;19:35. doi: 10.1186/s12935-019-0757-6 (PMC6377746; doi:10.1186/s12935-019-0757-6)
Supplement: Supplementary file 4 — Additional file 4. Synchronization of SiHa cells. Flow cytometry results for synchronization of SiHa cells. [file 12935_2019_757_MOESM4_ESM.pdf]

Additional file 4.

Contribution of voltage-gated sodium channel β-subunits to cervical cancer cells metastatic behavior

Ana Laura Sanchez-Sandoval, Juan Carlos Gomora*

Departamento de Neuropatología Molecular, División de Neurociencias, Instituto de Fisiología Celular, Universidad Nacional Autónoma de México. Ciudad de México, 04510, México.

*Corresponding author: jgomora@ifc.unam.mx

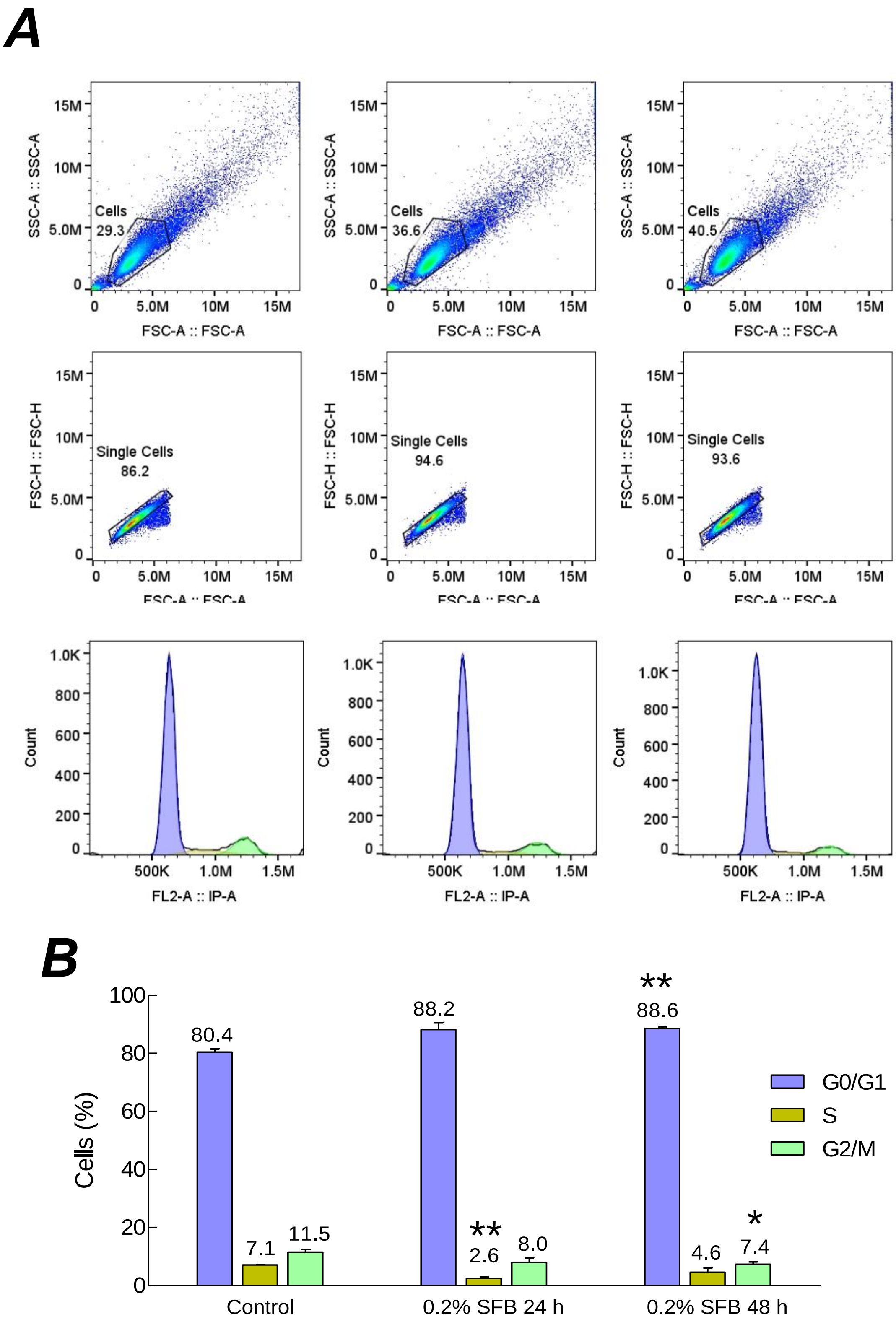
A
SSC-A :: SSC-A
FSC-A :: FSC-A
Cells
29.3
36.6
40.5
FSC-H :: FSC-H
Single Cells
86.2
94.6
93.6
Count
FL2-A :: IP-A
B
Cells (%)
80.4
7.1
11.5
88.2
**
2.6
8.0
**
88.6
4.6
*
7.4
G0/G1
S
G2/M
Control
0.2% SFB 24 h
0.2% SFB 48 h

Additional file 4. Synchronization of SiHa cells. ***A*)** Cell cycle synchronization in SiHa cells was analyzed by flow cytometry after incubation of cells with low-serum conditions (0.2% FBS) for 24 h (middle column) and 48 h (right column), comparing with control conditions (10% FBS; left column). Cells were gated using an FSC-A *vs* SSC-A dot-plot (upper panels); then, single cells were sub-gated using an FSC-A *vs* FSC-H dot-plot (second row) and finally cell cycle analysis was then performed on the FL2-A histogram using the Dean-Jett-Fox algorithm (third row). For each condition, at least 2×10^4 single cells were analyzed. Three independent experiments are summarized in the bar graphs (***B***) as means ± S.D. of the relative percentage of cells in each cell cycle phase. Significance: *$P < 0.05$, **$P < 0.01$, compared to control conditions.
