# Supplementary material for: Contribution of voltage-gated sodium channel β-subunits to cervical cancer cells metastatic behavior
Source: Cancer Cell Int. 2019 Feb 15;19:35. doi: 10.1186/s12935-019-0757-6 (PMC6377746; doi:10.1186/s12935-019-0757-6)
Supplement: Supplementary file 5 — Additional file 5. Analysis of NaVβ4 expression in CeCa biopsies and normal cervical tissue. Representative immunohistochemical images of cervical cancer biopsies and normal cervix tissues showing the absence of NaVβ4 in CeCa biopsies and the moderate expression of the protein in normal cervix. [file 12935_2019_757_MOESM5_ESM.pdf]

Additional file 5.

Contribution of voltage-gated sodium channel β-subunits to cervical cancer cells metastatic behavior

Ana Laura Sanchez-Sandoval, Juan Carlos Gomora*

Departamento de Neuropatología Molecular, División de Neurociencias, Instituto de Fisiología Celular, Universidad Nacional Autónoma de México. Ciudad de México, 04510, México.

*Corresponding author: jgomora@ifc.unam.mx

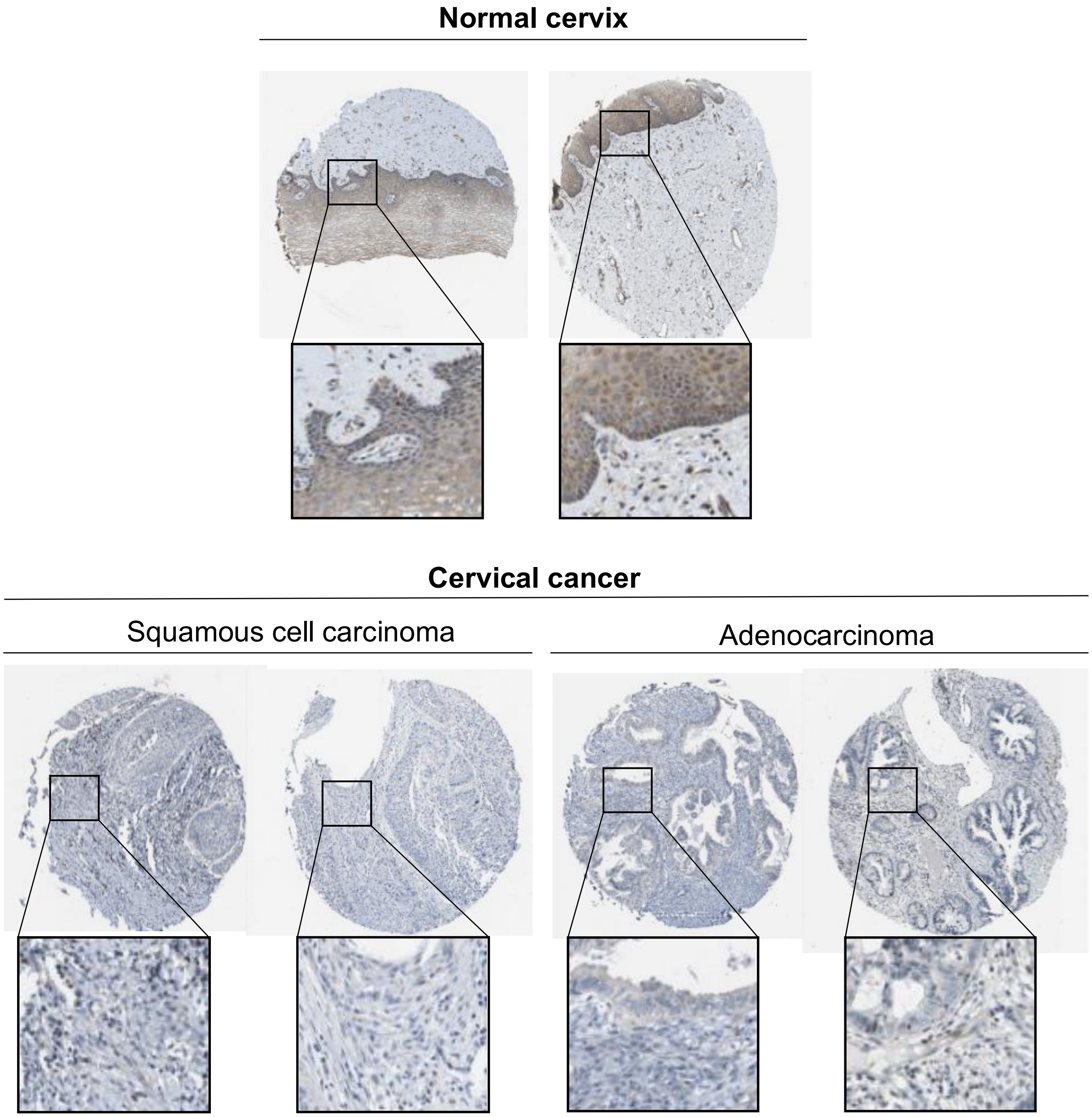

Additional file 5. Analysis of $Na_V\beta4$ expression in CeCa biopsies and normal cervical tissue. Representative immunohistochemical images of four cervical cancer biopsies (two squamous cell carcinomas and two adenocarcinomas) and two normal cervix tissues showing the absence of $Na_V\beta4$ in all the CeCa biopsies and the moderate expression of the protein in normal cervix. All the images were obtained from the Human Protein Atlas (http://www.proteinatlas.org/) (1,2). This database shows $Na_V\beta4$ staining of three different normal cervix biopsies, all of them

expressing moderate amounts of the protein, and twelve different CeCa biopsies, showing the lack of $Na_V\beta4$ signal in all of them, suggesting a correlation between the cervical cancer establishment and the downregulation of $Na_V\beta4$ expression.

1. Uhlen M, Fagerberg L, Hallstrom BM, Lindskog C, Oksvold P, Mardinoglu A, et al. Proteomics.Tissue-based map of the human proteome. Science. 2015; 347(6220):1260419.
2. Uhlen M, Oksvold P, Fagerberg L, Lundberg E, Jonasson K, Forsberg M, et al. Towards a knowledge-based Human Protein Atlas. Nat Biotechnol. 2010;28(12):1248-50.
